# NF-κB mediated enhancement of potassium currents by the chemokine CXCL1/growth related oncogene in small diameter rat sensory neurons

**DOI:** 10.1186/1744-8069-5-26

**Published:** 2009-05-28

**Authors:** Rui-Hua Yang, Judith A Strong, Jun-Ming Zhang

**Affiliations:** 1 Pain Research Center, Department of Anesthesiology, University of Cincinnati College of Medicine, Cincinnati, Ohio 45267-0531, USA

## Abstract

**Background:**

Inflammatory processes play important roles in both neuropathic and inflammatory pain states, but the effects of inflammation *per se *within the sensory ganglia are not well understood. The cytokine growth-related oncogene (GRO/KC; CXCL1) shows strong, rapid upregulation in dorsal root ganglion (DRG) in both nerve injury and inflammatory pain models. We examined the direct effects of GRO/KC on small diameter DRG neurons, which are predominantly nociceptive. Whole cell voltage clamp technique was used to measure voltage-activated potassium (K) currents in acutely cultured adult rat small diameter sensory neurons. Fluorescently labeled isolectin B4 (IB4) was used to classify cells as IB4-positive or IB4-negative.

**Results:**

In IB4-negative neurons, voltage-activated K current densities of both transient and sustained components were increased after overnight incubation with GRO/KC (1.5 nM), without marked changes in voltage dependence or kinetics. The average values for the slow and fast decay time constants at 20 mV were unchanged by GRO/KC. The amplitude of the fast inactivating component increased significantly with no large shifts in the voltage dependence of inactivation. The increase in K currents was completely blocked by co-incubation with protein synthesis inhibitor cycloheximide (CHX) or NF-κB inhibitors pyrrolidine dithiocarbamate (PDTC) or quinazoline (6-Amino-4-(4-phenoxypheny lethylamino;QNZ). In contrast, the voltage-activated K current of IB4-positive neurons was unchanged by GRO/KC. GRO/KC incubation caused no significant changes in the expression level of eight selected voltage-gated K channel genes in quantitative PCR analysis.

**Conclusion:**

The results suggest that GRO/KC has important effects in inflammatory processes via its direct actions on sensory neurons, and that activation of NF-κB is involved in the GRO/KC-induced enhancement of K currents.

## Background

Inflammatory processes are recognized to play key roles in chronic pain. The traditional distinction between inflammatory and nerve injury models of chronic pain has been recently augmented by the recognition that even nerve injury models have inflammatory components. Many cytokines and chemokines with previously established roles in the immune system have also been found to have direct effects on peripheral and central neurons, and to play key roles in pathologic pain [1-3]. One such chemokine is Growth-Related Oncogene (GRO/KC; systemic name CXCL1). We first became interested in this molecule because it was very strongly and rapidly upregulated in DRG in several different pain models, including the spinal nerve ligation model [4] and a model in which pain behaviors are evoked by localized inflammation of the DRG [5].

GRO/KC is well known for its role in neutrophil chemotaxis and degranulation early during inflammation. In this regard its effects are similar to those of other CXC family cytokines such as interleukin-8 (IL-8; CXCL8) in humans [6]. GRO/KC may also have direct roles in the nervous system, including roles in pathological pain. Both GRO/KC and its primary receptor, CXCR2 (IL-8Rb) are expressed in neurons and other cells in the central nervous system, under both normal and pathological conditions [7-13]. In the peripheral nervous system, GRO/KC stimulates calcium influx [14], and release of the pain-related peptide calcitonin gene-related peptide (CGRP) [15] from cultured neonatal DRG neurons. Levels of GRO/KC in inflamed muscle tissue correlate well with nociceptive behavior [16]. In general, these studies in peripheral nervous system suggest a pro-nociceptive role for GRO/KC (however, see [17]).

Previously we have described a rat pain model in which localized inflammation of the DRG (LID) is induced by depositing a small drop of the immune stimulator zymosan over the L5 DRG. This leads to prolonged mechanical pain behaviors, and a rapid increase in levels of GRO/KC and other pro-inflammatory cytokines [5] in the DRG. We have also demonstrated that LID causes marked increases in excitability, large increases in Na currents and, to a lesser degree, K currents [18] in small diameter DRG neurons as observed with patch clamp methods after acute culture. In that study, TTX-sensitive Na currents increased 2 to 3 fold in both IB4-positive and IB4-negative cells, while TTX-resistant Na currents increased over 2-fold but only in IB4-positive cells. Transient K currents increased over 2-fold, while sustained K currents showed a very modest though significant increase. The observed increases in Na and K current densities were due to increased amplitude, not to large shifts in voltage dependence of activation or inactivation; the increase in transient K current was due to increased amplitude of the faster-inactivating current of two kinetically distinct components. In a second study [19], we found that some of these effects on Na currents could be mimicked by overnight incubation with GRO/KC (1.5 nM). Overnight GRO/KC treatment in acutely cultured neurons led to increased excitability, and to 2- to 4 fold increases in TTX-resistant and TTX-sensitive Na currents (in both IB4-positive and IB4-negative cells) without altered voltage dependence or kinetic changes. Changes in Na current were blocked by a protein synthesis inhibitor, and we observed increases in mRNA abundance of particular Na channel isoforms that are already present in control cells. The results suggested that GRO/KC may have important pro-nociceptive effects through direct effects on neurons, and were consistent with the idea that some though not all of the changes observed after LID in vivo might be mediated by this chemokine. In the present study we have extended this line of research to study the in vitro effects of GRO/KC on voltage-activated K currents, as these were also increased by local DRG inflammation. We have also begun to examine possible signaling pathways by which GRO/KC might effect these changes in neuronal properties.

## Methods

### Animals

Young female Sprague-Dawley rats (body mass 100–150 g) were housed one or two -per cage under a controlled diurnal cycle of 12 h light and 12 h dark with free access to water and food. The ambient environment was maintained at constant temperature (22 ± 0.5°C) and relative humidity (60–70%). All the surgical procedures and the experimental protocol were approved by the institutional animal care and use committee of the University of Cincinnati (Cincinnati, Ohio, USA).

### Acute culture of sensory neurons

Rats were anesthetized by intraperitoneal injection of pentobarbital sodium (50 mg/kg). The bilateral L4 and L5 DRGs were isolated and the sheath was carefully removed in ice-cold normal Ringer solution. The connective tissue was digested by exposure to Ca^2+^-free solution containing 1.0% collagenase II (Fisher Scientific, Pittsburgh, Pennsylvania, USA) for 30 min at 37°C followed by washout in normal Ringer solution for another 10 min. DRGs were then dissociated by trituration with fire-polished Pasteur pipettes. DRG cells were plated onto poly-D-lysine coated glass coverslips in Medium199 (Sigma, St. Louis, Missouri, USA) containing 10% heat-inactivated FBS and 1000 U/ml each of penicillin and streptomycin. GRO/KC (rat recombinant, from Cell Sciences, Canton, Massachuesetts, USA and PeproTech, Rocky Hill, New Jersey, USA) and/or other drugs were added to the medium immediately after plating or as indicated, and the DRG cells were incubated at 37°C (5% CO_2 _balance air). Recordings were made after overnight culture and drug incubation (16–24 hours), and cells were selected for recording based on diameter (< 25 μm) and the absence of processes. Isolectin B4 (IB4) 1 μg/μl (conjugated to FITC; Sigma) was added to the culture medium for 30–60 min prior to the start of recording sessions to identify IB4-postive (primarily non-peptidergic) and IB4-negative (primarily peptidergic) neurons [20,21]. This procedure has been previously shown not to affect the currents measured [22]. Neurons showing a robust fluorescence signal were classified as IB4-positive and those displaying no signal at all, IB4-negative. We have previously shown that GRO/KC incubation does not alter the fraction of cells that is IB4-positive [19]; hence in some cases IB4-status was determined before recording from a cell in order to obtained matched sets of data. Throughout each individual experiment, we alternated between recording from control and experimental cells during the recording period so as to compare control and experimental cells taken from the same cultures. For experiments in which there were four experimental groups (e.g. GRO/KC, GRO/KC plus drug, control, control plus drug), only two groups were examined per culture. Some sets of cultures were used to determine the effects of drug on GRO/KC treated cells, and different sets were used to determine the effects of the drug on control cells in the absence of GRO/KC.

Drugs were applied at the following concentrations: GRO/KC 1.5 nM, Pyrrolidine dithiocarbamate (PDTC, Sigma) 50 μM, Quinazoline (QNZ, Calbiochem) 100 nM (stocks made with ethanol and control cells treated with vehicle), cycloheximide (Sigma), 3.55 μM.

### Electrophysiological recording

After over-night culture (16–24 hours), coverslips were transferred to a recording chamber. Whole cell voltage-clamp recordings of small DRG neurons (diameter 15–25 μm) were conducted at room temperature with an AxoPatch-200B amplifier (Molecular Devices Corp, Union City, California, USA). Patch pipettes (2.5–4.0 MΩ) were fabricated from borosilicate glass. Data were acquired on a Pentium IV computer with the Clampex 8 program (Molecular Devices Corp). The cell capacitance artifact was canceled by the nulling circuit of the recording amplifier. Voltage errors were minimized by using ≥ 80% series resistance compensation. The current was filtered at 5 kHz and sampled at 50 kHz. The recording chamber was continuously perfused at room temperature with oxygenated bath solution at a flow rate of 1–2 ml/min.

Resting membrane potential was measured 1 min after a stable recording was obtained. Current pulses from -0.2 to 0.4 nA (80-ms pulse duration) were delivered in increments of 0.03 nA until one or more APs were evoked. Neurons were selected for further study if they had a resting membrane potential that was more negative than -45 mV and if they exhibited overshooting action potentials. The bath solution was then switched to that used for measuring K current (see below). Total K current was measured by depolarizing voltage steps after a 1-s prepulse to -120 mV. The difference between the peak current and the current at the end of the pulse was used as a measure of the transient K current, and the current at the end of the pulse was used as a measure of the sustained current. Voltage dependence of inactivation was measured by test pulses to 20 mV after 1-s prepulses to voltages between -100 and 0 mV.

### Solutions for recording potassium currents

The normal bath solution contained 130 mM NaCl, 5 mM KCl, 2 mM CaCl_2_, 1 mM MgCl_2_, 10 mM HEPES, and 10 mM glucose. The pH was adjusted to 7.4 with NaOH, and the osmolarity was adjusted to approximately 300–310 mOsm with sucrose. After the whole cell configuration was established and current clamp measurements of excitability and resting potential were completed, the bath solution was changed to one used for recording K currents, which contained 130 mM choline Cl, 5 mM KCl, 1 mM MgCl_2_, 2 mM CoCl_2_, 10 mM HEPES, and 10 mM glucose. The pH was adjusted to 7.4 with Tris base, and the osmolarity was adjusted to approximately 300–310 mOsm with sucrose. The pipette solution contained 140 mM KCl, 1 mM CaCl_2_, 2 mM MgCl_2_, 11 mM EGTA, 10 mM HEPES, 2 mM Mg adenosine triphosphate, and 1 mM Li guanosine triphosphate. The pH was adjusted to 7.2 with Tris base, and osmolarity was adjusted to approximately 290–300 mOsm with sucrose. Voltages were not corrected for liquid junction potentials, which were estimated to be less than 10 mV in all cases.

### Data analysis and statistics

Data were analyzed using Clampfit 9 (Molecular Devices Corp), Graphpad Prism (GraphPad Software, Inc., San Diego, California, USA), Origin 7 (Origin Lab Corp., Northampton, Massachusetts, USA), and SigmaStat (Systat Software, Inc., San Jose, California, USA). Currents were normalized by cell capacitance. Data are expressed as mean ± standard error of the mean (SEM). Statistical significance of differences between average values in experimental and control neurons was analyzed by Student's t-test or, for data that were not normally distributed, the Mann-Whitney rank sum test. In the case of multiple comparisons over a voltage range for activation or inactivation data, the data were analyzed by two-way repeated measures ANOVA (RM ANOVA), with pairwise multiple comparison (Holm-Sidak method) to determine at which voltages the differences between experimental and control cells were significant if an overall effect of GRO/KC or drug treatment was observed. Significance was ascribed for P < 0.05.

### Quantitative PCR

Total RNA was isolated from DRG cultures under conditions similar to those used in electrophysiological recordings, using a Stratagene Absolutely RNA Microprep Kit (Stratagene, La Jolle, California. USA). Immediately after isolation, RNA was reverse transcribed into cDNA using an iScript cDNA kit (Bio-Rad, Hercules, California USA). Cells treated overnight with 1.5 nM GRO/KC were compared with control cells from the same animal. Relative channel expression was determined by quantitative PCR using a Stratagene MX-Pro 3005P. cDNA samples were amplified in triplicate (10 min at 95°C, followed by 40 cycles of 30 s 95°C, 60 s 60°C, then 60 s 72°C, followed by a 60 s step to 76°C for SYBR green fluorescence measurement) in a 25 μl reaction volume (400 nM each forward and reverse primers, SYBR green reaction mix (Roche Applied Science, Indianapolis, Indiana, USA)). Fluorescence was normalized with the reference dye ROX, and gene expression was normalized to the housekeeping gene hypoxanthine ribosyltransferase (HPRT; [23]), run on the same plate. Primers were selected using the Primer3 program [24] and were designed to include exon-intron boundaries to minimize contamination by genomic DNA, except for the intronless Kv1.3. Primers were examined with an in silico virtual PCR program to ensure that no other products were predicted from rat cDNA or genomic DNA , and/or by blasting the predicted PCR products against the rat reference mRNA sequence database. PCR products were verified by melting point analysis at the end of each experiment, and, during protocol development, by gel electrophoresis. Template from control cells was run side-by-side with template from GRO/KC-treated cells from the same culture. The baseline calculation, threshold, threshold cycle, and efficiencies of the amplification reactions were estimated directly from the reference-dye normalized amplification plots using the LinReg program and a correction for efficiencies was included in the relative expression calculations [25]. Efficiencies were generally similar for control and GRO/KC treated samples, and across experiments; a single efficiency value was used for control and GRO/KC samples for each gene. Forward and reverse primer sequences used for the genes listed in Table 1[26] are available in Additional file 1.

**Table 1:** Quantitative PCR measurements of selected voltage-gated K channels

Channel	Type*	Rat gene symbol	Rat Gene ID	Abundance in control cells relative to **Kv 1.1**	Fold-change w/GRO/KC
Kv 1.1	DR	*Kcna1*	[GenBank: 24520]	1.00 ± 0.17	1.18 ± 0.12
Kv 1.2	DR	*Kcna2*	[GenBank: 25468]	0.25 ± 0.04	1.24 ± 0.19
Kv 1.3	DR	*Kcna3*	[GenBank: 29731]	0.06 ± 0.04	0.81 ± 0.23
Kv 1.4	A-type	*Kcna4*	[GenBank: 25469]	0.16 ± 0.11	0.82 ± 0.22
Kv 3.1	DR	*Kcnc1*	[GenBank: 25327]	0.02 ± 0.00	1.14 ± 0.20
Kv 3.4	A-type	*Kcnc4*	[GenBank: 684516]	0.05 ± 0.00	1.11 ± 0.09
Kv 4.3	A-type	*Kcnd3*	[GenBank: 65195]	0.06 ± 0.01	0.89 ± 0.05
MinK-2	accessory	*Kcne3*	[GenBank: 63883]	0.02 ± 0.00	0.98 ± 0.15

N = 4 cultures from 4 animals, each of which provided a pair of RNA samples (control cells and GRO/KC treated cells). All expression values were normalized to that of the housekeeping gene HPRT from the same sample; fold changes (GRO/KC/control) were calculated for each individual pair of matched samples. For comparison of relative abundance of channel types in control cells, expression data were normalized to the most abundant channel observed, Kv1.1. None of the fold-change values after overnight GRO/KC incubation was significantly different from 1.

*, channel type as given by reference [26], "DR" = delayed rectifier; however, K channel properties may also depend on accessory proteins and heteromer composition.

## Results

### Incubation with GRO/KC enhances voltage-gated K currents in IB4-negative cells

**Figure 1:**
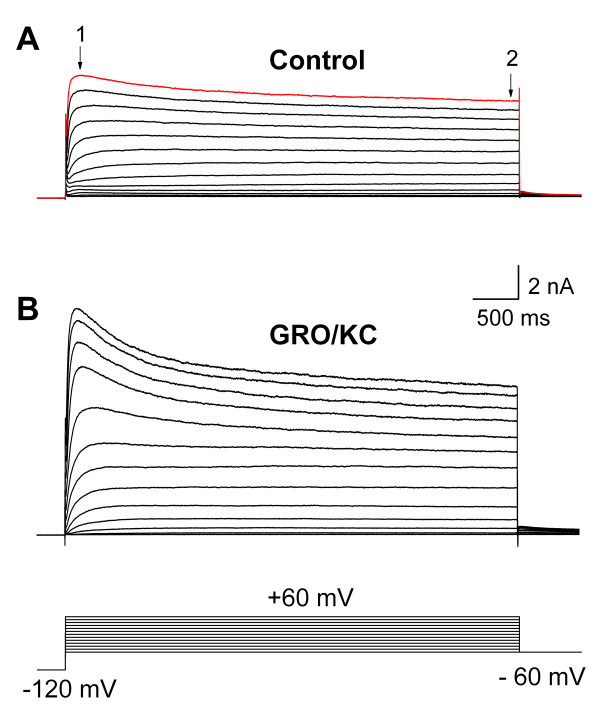
**Voltage activated K currents of DRG neurons in IB4-negative cells**. Currents shown were evoked by depolarizing pulses from -60 to + 60 mV. Examples are shown from a control cell (A) and from a GRO/KC incubated cell (B). The difference between the peak current (1) and the current at the end of the pulse (2) was used as a measure of the transient K current.

Voltage-activated K currents were recorded as shown in Figure 1. The membrane was held at -60 mV, and voltage steps were applied in 10 mV increments up to a value of +60 mV after a 1s prepulse to -120 mV. The total K current was comprised of sustained components, as measured at the end of the 500-ms pulse, and transient components which largely decayed during the pulse. In view of the complexity and variability of K currents in DRG neurons [27-29], and for comparison with our previous studies [18,19], we analyzed the effects of GRO/KC incubation on these two components, the transient (IA) and the sustained (IK). The transient component did not always completely decay during the 500 ms pulse; however, we determined that estimating the steady state (sustained) current from the extrapolated constant value of two-exponential fits to the falling phase of the current resulted in only a minor correction in most cells, and did not change the overall results reported here (data not shown; see [18]).

**Figure 2:**
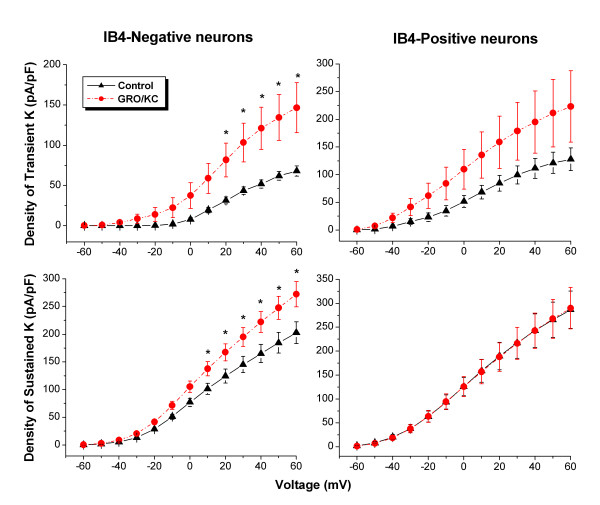
**Voltage activated K current in IB4-negative dorsal root ganglion neurons increased after GRO/KC incubation**. The current densities of both the transient component and sustained components were increased after overnight incubation with GRO/KC (1.5 nM) in IB4-negative neurons. *, the effect of GRO/KC was significant at all points above 10 mV (left). There was no significant effect in IB4-positive neurons (right). Data are from 5 cultures; in IB4-negative neurons, N = 15 GRO/KC and 16 control cells; in IB4-Positive neurons, N = 16 GRO/KC and 15 control cells.

**Figure 3:**
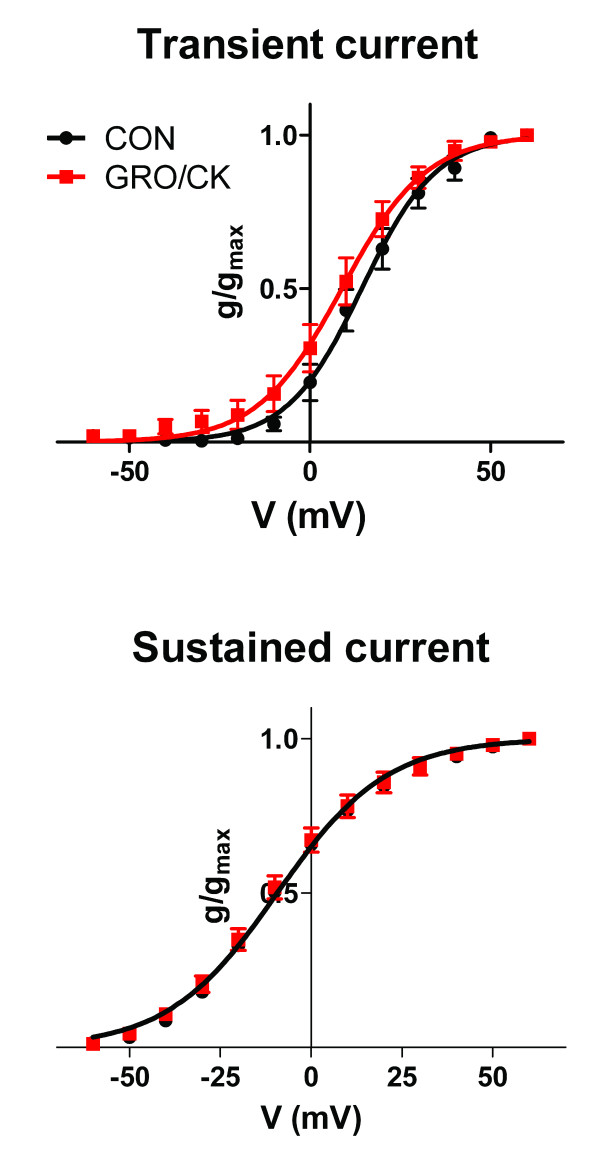
**Effect of GRO/KC treatment on voltage-dependence of K current activation in IB4-negative cells**. Data from Figure 2 were converted to conductance values using the calculated value of the K Nernst potential. Data were fitted with the Boltzmann equation. Top: the transient component showed a small but significant leftward shift of V_1/2_, from 14.3 mV in control cells to 8.8 mV in GRO/KC treated cells (p < 0.0005). The Hill slope values were 10.3 and 11.7 mV, respectively (for e-fold change; difference not significant). Bottom: there was no significant change in the voltage dependence of activation of the sustained component. Best-fit shared parameters were -9.3 mV for V_1/2 _and 15.1 mV for Hill slope.

The current densities of both transient (P < 0.001) and sustained components (P < 0.001) were increased after overnight incubation with GRO/KC (1.5 nM) in IB4-negative neurons (Figure 2). Two-way repeated-measures analysis of variance indicated that the effect of GRO/KC was significant at all points above 10 mV in IB4-negative cells. These effects were primarily due to increased amplitude of the current, without any large changes in the voltage dependence of activation (Figure 3); however, the activation curve for the transient component showed a small (5.5 mV) but significant leftward shift after GRO/KC incubation.

There was no significant change in K current density in IB4-positive neurons after GRO/KC incubation (Figure 2). In subsequent figures we show results only from IB4-negative neurons. However, all experiments shown were also conducted in IB4-positive neurons. These experiments simply confirmed the original findings; we did not observe significant differences due to GRO/KC or any drug treatment between any of the experimental groups in IB4-positive cells. The apparently greater density of the transient and sustained currents in control IB4-positive cells compared to control IB4-negative cells (Figure 2) was not statistically significant.

In the course of these experiments we also replicated our previously published results [19] showing that GRO/KC incubation led to decreased rheobase and lowered action potential threshold in IB4-negative cells, and that the cells acquired the ability to fire repetitively throughout the course of a prolonged current injection.

**Figure 4:**
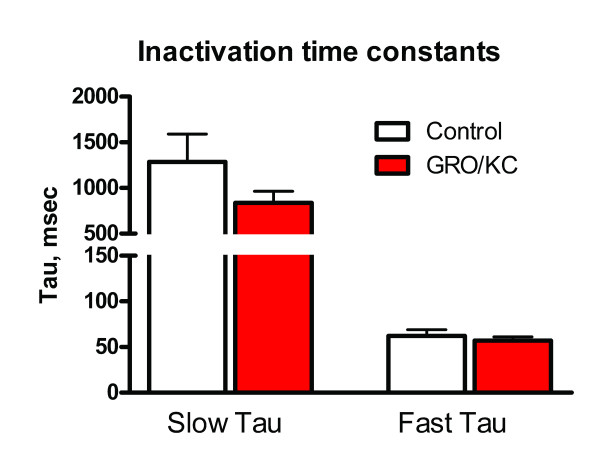
**Inactivation time constants at +20 mV are not affected by GRO/KC incubation**. The falling phase of the currents at a test pulse of +20 mV following prepulses between -100 and 0 mV was fitted with the sum of two exponentials. The time constants for the slow and fast components were not significantly changed by GRO/KC incubation.

The voltage dependence of steady state inactivation was also determined for the voltage-activated K currents. The protocol consisted of a 1-s conditioning prepulse to potentials ranging between -100 and 0 mV followed by a voltage step to a +20 mV test pulse for 1 s. As in our previous study [18], the decay of the outward current at +20 mV could be well described in most cells as the sum of a sustained component and two exponentially decaying transient components whose time constants differed by an order of magnitude. The average values for the slow and fast time constants in IB4-negative cells were unchanged by GRO/KC (1100.1 ± 254 ms.1 vs. 861.3 ± 154.2 ms, and 62.4 ± 7.8 ms vs. 57.6 ± 4.8 ms, respectively; Figure 4). Fast and slow time constants in IB4-positive cells were also not significantly changed by GRO/KC incubation, and did not differ significantly from those observed in IB4-negative cells.

**Figure 5:**
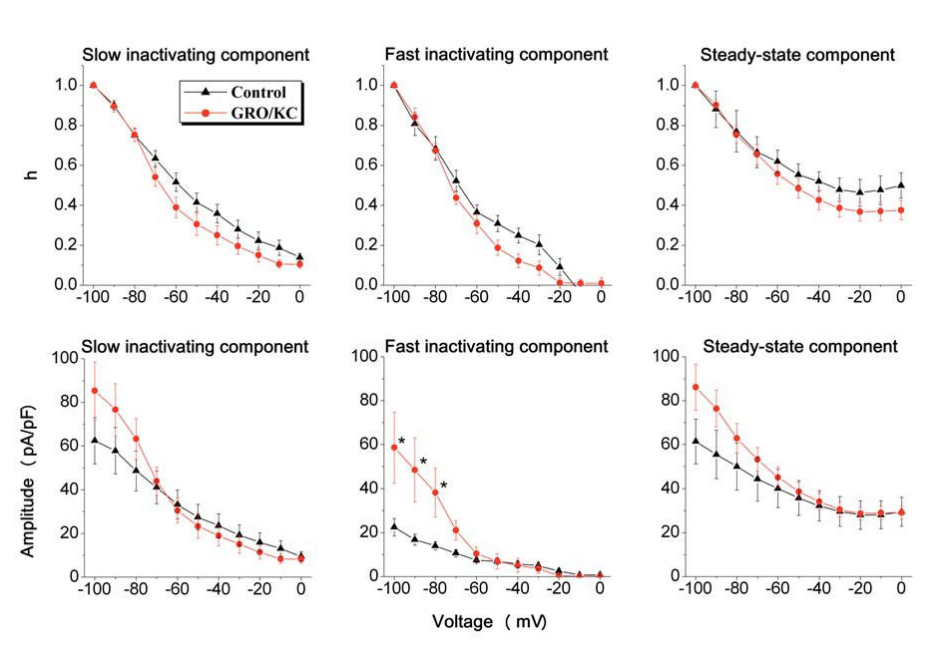
**Effects of GRO/KC on the fitted two exponential fits to steady state inactivation data**. The falling phase of currents during a pulse to +20 mV after a prepulse to the indicated voltage was fit with the sum of two exponentials plus a steady state value. At this test voltage, most cells show two kinetically distinct components of the transient K current. The values of the amplitudes of each component are normalized to the value at -100 mV (top): left, slow inactivating; middle, fast inactivating; right, steady state component. The bottom panels show the corresponding absolute values of the fitted amplitudes. * Individual voltages at which the differences between GRO/KC and control values were significant. Data in this and previous figures are from 5 cultures; N = 12 GRO/KC cells and 11 control cells.

The steady state inactivation of each of these three components was studied individually by fitting the data at the +20 mV test pulse with the sum of two exponentials plus a constant (steady state) value, as in our previous study [18]. As shown in Figure 5, in IB4-negative cells the fast inactivating (middle panel) component showed a simple increase in amplitude after GRO/KC (*P *= 0.04), with no significant shift in the voltage dependence of inactivation. For the slow inactivating component and steady state component (Figure 3), the amplitudes were numerically increased, but this difference was not significant (*P *= 0.71 and 0.44, respectively), and the voltage dependence was not affected.

### Enhancement of K currents by GRO/KC is blocked by a protein synthesis inhibitor

**Figure 6:**
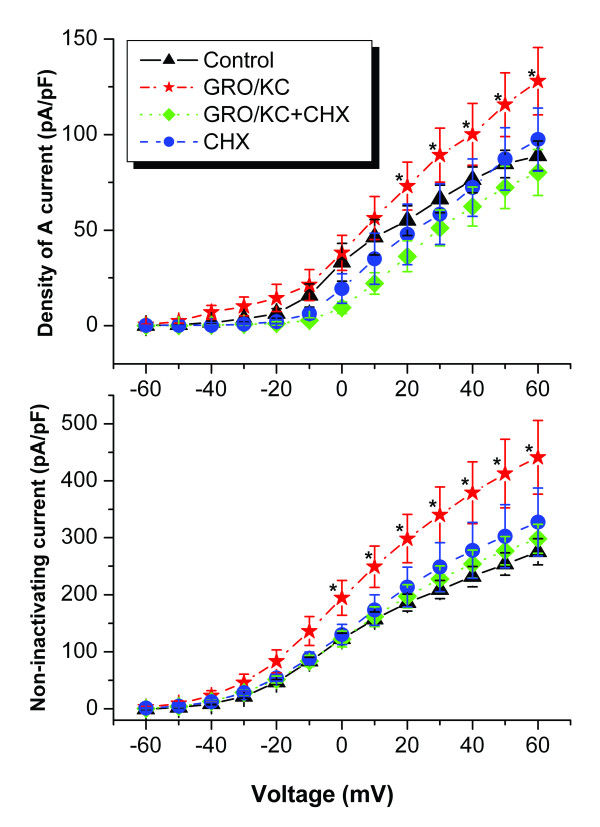
**Treatment with the protein synthesis inhibitor cycloheximide (CHX, 3.5 μM) blocked the effects of GRO/KC on K currents in IB4-negative cells**. CHX had no effect on K currents in control cells (P = 0.755). CHX was added at the same time as GRO/KC and was present throughout the incubation period. GRO (N = 19) and GRO+CHX (N = 18) cells were from 9 cultures; 12 control cells and 8 CHX cells were from 2 cultures.

The finding that GRO/KC enhanced the magnitude of K currents in IB4-negative cells, without marked shifts in voltage dependence of activation or kinetics suggested that the primary effect might be an increased number of K channels, and hence that protein synthesis might be required. To test this idea, we conducted experiments using the protein synthesis inhibitor cycloheximide. The results are shown in Figure 6. In general, cycloheximide prevented the GRO/KC-induced increases in K current density, without significantly affecting baseline current densities (P = 0.755). In IB4-negative cells, the only significant differences observed were between the GRO/KC treated groups and all other groups.

### Effects of GRO/KC are blocked by NF-κB inhibitors

**Figure 7:**
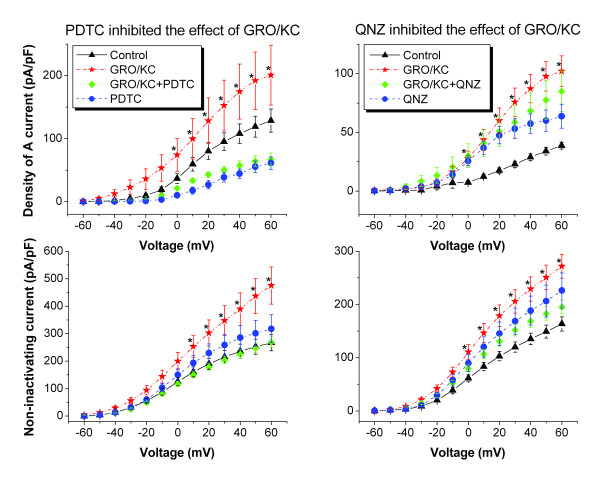
**The NF-κB inhibitors pyrrolidine dithiocarbamate (PDTC) and quinazoline (6-Amino-4-(4-phenoxypheny lethylamino), QNZ) blocked the effects of GRO/KC on K currents**. QNZ was dissolved in Ethanol. PDTC or QNZ was added 1 h before GRO/KC and was present throughout the incubation period. Neither PDTC nor QNZ had an effect on K currents in control cells (P = 0.867 and 0.999, respectively). *, significantly different from all other groups at this voltage. In PDTC experiments, GRO/KC (N = 18 cells), GRO/KC+PDTC (N = 18) were from 4 cultures, control (N = 13) and PDTC (N = 11) were from 4 cultures; in QNZ experiments, GRO/KC (N = 26), GRO/KC+QNZ (N = 26) were from 7 cultures, control (N = 6) and QNZ (N = 9) were from 2 cultures.

Two NF-κB inhibitors, pyrrolidine dithiocarbamate (PDTC) and quinazoline (6-Amino-4-(4-phenoxypheny lethylamino), QNZ) were used to explore the possible signaling pathway of GRO/KC effects on K currents. PDTC or QNZ was added 1 h before GRO/KC and was present throughout the incubation period. Both PDTC and QNZ blocked the effects of GRO/KC on K currents. Neither had effect on K currents in control cells (P = 0.867 and 0.999, respectively) (Figure 7).

### Quantitative PCR analysis of selected K channels

The electrophysiological experiments suggested that the increase in K current in IB4-negative cells after GRO/KC incubation was not due to the appearance of new channels with novel properties not seen in control cells. Qualitatively consistent with this idea, quantitative PCR experiments on selected voltage activated K channels did not reveal any types that were present only after GRO/KC analysis (Table 1). However, we did not examine all the possible genes coding for K currents that might mediate the delayed rectifier or A-type currents observed.

## Discussion

In this study we found that overnight incubation with GRO/KC (CXCL1), a chemokine that is rapidly upregulated in the DRG in several different models of chronic pain, caused upregulation of K current amplitudes in acutely isolated small diameter IB4-negative DRG neurons in short-term culture. This upregulation required protein synthesis and activation of NF-κB. Many studies on the role of chemokines in pain conditions, including GRO/KC (e.g., [30]), have focused on their indirect effects on neurons, via stimulation of release of inflammatory mediators from immune and glial cells that then act on sympathetic and sensory neurons. Since our cultures are relatively sparse, and we record primarily from isolated neurons with few or no glial cells attached, it seems most likely that the effects of GRO/KC incubation on K currents were due to direct effects on the neurons. Our previous study indicated that GRO/KC receptors are found on both IB4-positive and IB4-negative neurons in acute culture (and in vivo), and that GRO/KC can have important pro-nociceptive effects via its direct actions on sensory neurons [19].

We observed effects on K currents at a relatively low GRO/KC concentration of 1.5 nM, which is well within the physiological range for most peptide receptor actions and within the range of concentrations reported to be effective at the CXCR2 receptor [15]. This concentration was chosen based on our previous study, in which we demonstrated that incubation with 1.5 nM GRO/KC but not lower concentrations (0.06 or 0.28 nM) caused significant upregulation of Na currents.

Potassium currents have important roles in modulating neuronal excitability. Previous studies described three major components of voltage-gated potassium current in small sensory neurons, including non-inactivating current and fast and slow transient currents as we have analyzed here [27,29,31-33]. However, this is undoubtedly an oversimplification; for example, one study delineated six components of K current, five of which were in small diameter neurons such as studied here [28]. K channel genetic diversity suggests a possibly larger number, especially given the existence of functionally distinct heteromers and splice variants in the several gene families that mediate voltage-activated K currents [34]. Our preliminary quantitative PCR study indicated that all seven of the delayed rectifier and A-type (inactivating) channels as well as the regulatory accessory protein MinK-P2 that we selected for study were readily observed in our acute DRG cultures. The method was not sensitive enough to detect upregulation of particular channels that might account for our electrophysiological results, though this is perhaps not surprising given the relatively small increase that would be expected (2-fold or less increases in current density occurring in less than half of the cells present in the cultures). The expression results confirmed that, qualitatively consistent with the electrophysiological findings, GRO/KC incubation did not induce marked upregulation of a K channel gene with distinct properties that was absent in control cells. However, it should be noted that we looked at only 8 genes, not every gene known to possibly mediate the types of K currents measured.

The most robust effect of GRO/KC in this study was the relatively large increase (aprox. 2 fold) in amplitude of the faster-inactivating component of the transient K current. (This may correspond to I_Af _and/or I_ht _in the paper by Gold et al., given that our measurements were at +20 mV where these components have similar decay time constants.) The complex forms of the averaged steady state inactivation curves we observed even when dividing the K current into fast-inactivating, slow-inactivating, and constant components (Fig 5) suggest that multiple channel types are present. Within the context of the simplified analysis used here, the effect on the transient K current is qualitatively quite similar to that observed in our previous study of the effects on localized DRG inflammation on K currents ([18]; see Introduction). However, that study did not divide cells into IB4-positive and IB4-negative cells, yet still showed over 2 fold increases in magnitude of the faster inactivating component, whereas in the present study a similar increase was observed in IB4-negative cells with no increase in IB4-positive cells. Since IB4-negative cells comprise about half of the population of small cells under our culture conditions, it seems that the observed effects of GRO/KC are consistent with the effects of localized inflammation on transient K currents, but are not sufficient to account for all those effects quantitatively.

In contrast, the effects of GRO/KC incubation on sustained K currents were much more modest, and unlikely to play a role at physiological voltages. This increase was significant in IB4-negative cells when currents were examined from a -120 mV prepulse (Fig 2), but not from more positive prepulses going into the physiological range (Fig 5). These effects on sustained K current are very similar to those observed after LID [18]. Calcium-activated K currents were not examined in the current study because of the presence of EGTA in the recording pipette and Co^2+ ^replacing Ca^2+ ^in the extracellular solution.

Potassium channel reduction is thought to contribute to the increased excitability and generation/patterning of spontaneous activity in sensory neurons following peripheral nerve injury [35-37]. Potassium currents are not always reduced under pathological conditions; they may be upregulated as recently described by our group in a pain model induced by localized inflammation of the DRG [18]. In that model, both potassium currents and sodium currents are increased in small DRG neurons after a localized inflammation, and the increase in Na current density is quite large (2-fold or more). It seems likely that it is the balance of effects on Na and K (and other currents) which determines the net effect on excitability.

Our previous study of effects of GRO/KC incubation on excitability parameters [19] showed that many measures of excitability were increased by GRO/KC in both IB4-negative and IB4-positive cells. However, two differences between the two subtypes were that IB4-negative cells showed a more marked increase in the ability to fire repetitively, as well as an increase in the after-hyperpolarization. Hence it is interesting that only these IB4-negative cells showed an increase in transient K currents, which can play a role in making repetitive firing possible [38,39]. However, it should be noted that other currents not examined in this study may also play a role in determining the afterhyperpolarization and repetitive firing, such as the hyperpolarization-activated current IH [40], calcium-activated K-currents [41], and the kinetic properties of the various Na channel subtypes [42].

The protein synthesis inhibitor cycloheximide (CHX) completely blocked the effects of GRO/KC on K currents, which indicated that protein synthesis is required for the effect on K currents. The simplest explanation would be that additional K channels are synthesized after GRO/KC incubation, as this would be consistent with the observation that primarily it is the magnitudes of some components of the K currents are altered, not their activation ranges or decay time constants. However, there may be other possibilities such as synthesis of proteins that modulate channels already present. GRO/KC actions are thought to be mediated by the CXCR2 receptor, a G-protein-coupled receptor, which has been shown to activate a number of different signaling pathways in neurons, including intracellular Ca^2+^, inositol tris-phosphate, MAP kinases, and CREB [43]. All of these pathways can lead to enhanced transcription and protein synthesis through a number of signal transduction pathways.

Nuclear factor kappa B (NF-κB) is a transcription factor which serves as a transducer between extracellular signals and gene expression. We chose this pathway for initial study because of its key role in inflammation, and showed that pretreatment with either of two NF-κB inhibitors, PDTC and QNZ, prevented the enhancement of K currents induced by GRO/KC. A number of proinflammatory cytokines, including TNF-α [44], IL-1α [45], IL-6 and ciliary neurotrophic factor [46], can activate NF-κB in neurons leading to the altered expression of target genes and in some cases blocking the apoptosis pathway. CXCR2, which is the primary GRO/KC receptor in neurons, has also been shown to activate the NF- κB pathway in cardiac endothelial cells [47]. To our knowledge, this is the first report of CXCR2 receptor using this pathway in neurons. Pannaccione et al [48] demonstrated that NF-κB inhibitors block the increase in mRNA and protein expression of KV3.4 and its regulatory accessory protein MinK-related peptide 2, as well as the increase in corresponding K currents, that are triggered by Aβ peptide exposure in both PC-12 cells and hippocampal neurons.

## Conclusion

On the whole, our data suggest that GRO/KC (CXCL1), which markedly increases in DRG during the early phase of several models of pathological pain, has important effects in inflammatory processes via its direct actions on sensory neurons, and that activation of NF-κB is involved in the GRO/KC-induced enhancement of K currents.

## Competing interests

The authors declare that they have no competing interests.

## Authors' contributions

RY conducted the patch clamp experiments. RY drafted the manuscript. RY and JAS participated in qPCR experiments, data analysis, statistical analysis, and experimental design. JAS helped to draft the manuscript. JZ conceived of the study, and participated in its design and coordination and helped to draft the manuscript. All authors read and approved the final manuscript.

## Supplementary Material

Additional file 1 **Sequences of Primers used in qPRC experiments**. Contains information about the primers used for the qPCR experiments. Click here for file
